# Accuracy and determinants of perceived HIV risk among young women in South Africa

**DOI:** 10.1186/s12889-017-4593-0

**Published:** 2017-07-21

**Authors:** Brendan Maughan-Brown, Atheendar S. Venkataramani

**Affiliations:** 1 Southern Africa Labour and Development Research Unit, University of Cape Town, Cape Town, South Africa; 2 Department of Medical Ethics and Health Policy, Perelman School of Medicine, University of Pennsylvania, Blockey Hall, #1102, 423 Guardian Dr, Philadelphia, PA 19104 USA

**Keywords:** Risk perceptions, South Africa, HIV testing, Sexual behaviour, Women, Stigma

## Abstract

**Background:**

HIV risk perceptions are a key determinant of HIV testing. The success of efforts to achieve an AIDS-free generation – including reaching the UNAIDS 90–90-90 target – thus depends critically on the content of these perceptions. We examined the accuracy of HIV-risk perceptions and their correlates among young black women in South Africa, a group with one of the highest HIV incidence rates worldwide.

**Methods:**

We used individual-level longitudinal data from the Cape Area Panel Study (CAPS) from 2005 to 2009 on black African women (20–30 years old in 2009) to assess the association between perceived HIV-risk in 2005 and the probability of testing HIV-positive four years later. We then estimated multivariable logistic regressions using cross-sectional data from the 2009 CAPS wave to assess the relationship between risk perceptions and a wide range of demographic, sexual behaviour and psychosocial covariates of perceived HIV-risk.

**Results:**

We found that the proportion testing HIV-positive in 2009 was almost identical across perceived risk categories in 2005 (no, small, moderate, great) (*χ* ^*2*^ = 1.43, *p* = 0.85). Consistent with epidemiologic risk factors, the likelihood of reporting moderate or great HIV-risk perceptions was associated with condom-use (aOR: 0.57; 95% CI: 0.36, 0.89; *p* < 0.01); having ≥3 lifetime partners (aOR: 2.38, 95% CI: 1.53, 3.73; *p* < 0.01); knowledge of one’s partner’s HIV status (aOR: 0.67; 95% CI: 0.43, 1.07; *p* = 0.09); and being in an age-disparate partnerships (aOR: 1.73; 95% CI: 1.09, 2.76; *p* = 0.02). However, the likelihood of reporting moderate or great self-perceived risk did not vary with sexually transmitted disease history and respondent age, both strong predictors of HIV risk in the study setting. Risk perceptions were associated with stigmatising attitudes (aOR: 0.53; 95% CI: 0.26, 1.09; *p* = 0.09); prior HIV testing (aOR: 0.21; 95% CI: 0.13, 0.35; *p* < 0.01); and having heard that male circumcision is protective (aOR: 0.38; 95% CI: 0.22, 0.64; *p* < 0.01).

**Conclusions:**

Results indicate that HIV-risk perceptions are inaccurate. Our findings suggest that this inaccuracy stems from HIV-risk perceptions being driven by an incomplete understanding of epidemiological risk and being influenced by a range of psycho-social factors not directly related to sexual behaviour. Consequently, new interventions are needed to align perceived and actual HIV risk.

**Electronic supplementary material:**

The online version of this article (doi:10.1186/s12889-017-4593-0) contains supplementary material, which is available to authorized users.

## Background

In recent years, there has been increasing optimism around achieving an AIDS-free generation [1]. The Joint United Nations Programme on HIV/AIDS has outlined an ambitious blueprint to do so: ensuring that 90% of persons living with HIV/AIDS (PLWHA) know their serostatus; initiating 90% of these individuals on treatment; and achieving viral load suppression in 90% of this group [2]. Successfully reaching these targets will require bolstering the entire cascade of HIV care, which, in turn, starts with generating demand for HIV testing services [3, 4].

Across a wide range of population groups and settings, HIV-risk perceptions influence demand for HIV testing [5–10]. Incorrect risk perceptions may therefore impede efforts to diagnose and treat afflicted individuals. For example, low perceived risks of HIV infection may hinder efforts to diagnose cases in areas where seroprevalence is high. However, there is limited evidence around the accuracy of HIV risk perceptions – that is, the extent to which stated perceptions of future HIV risk correctly predict future HIV status. A small literature illustrates contexts where average HIV risk perceptions are at odds with actual disease prevalence [11, 12], and therefore suggests inaccuracies in self-perceived HIV risk. Potential drivers of inaccurate risk perceptions may include an incomplete consideration of the riskiness of sexual behaviors or the influence of factors outside of direct HIV risk factors [11, 13–18]. However, no single study has systematically examined the influence of these diverse factors.

Accordingly, it is imperative to understand the accuracy of HIV risk perceptions better, and - if these are inaccurate – to understand how such perceptions are formed, particularly in high-risk populations. In this study, we used longitudinal data to prospectively examine the accuracy of HIV risk perceptions among young black women in South Africa, a group that faces a significant burden of disease from HIV/AIDS. Peak prevalence rates exceed 30% (for women aged 30–34), with an incidence rate of approximately 4.5% (for women aged 20–34) [19, 20]. We then examined the correlates of perceived HIV risk, expanding on the literature by using measures both of sexual behaviour and psychosocial factors.

## Methods

### Data

We used data from the Cape Area Panel Study (CAPS) [21]. The first wave of CAPS, conducted in 2002, surveyed a representative sample of 4752 young adults (ages 14 to 22) using a two-stage sample stratified by race. In the first stage, primary sampling units (PSUs) were selected based on the 1996 Census enumeration areas, and in the second stage households were randomly selected from PSUs. Respondents were re-interviewed (face-to-face in participants’ first language) up to four more times. The last wave was fielded in 2009, at which time respondents were 20–30 years old. Written consent was obtained from all respondents, and written parental consent was obtained for interviews with respondents under the age of 18.

Our study focused on the sample of black women, given the elevated risk of HIV infection in this sub-population [19]. The initial 2002 CAPS sample comprised 1221 black women of whom 833 (68%) and 746 (61%) were re-interviewed in 2005 and 2009 respectively. Extensive face-to-face questionnaires were administered in each wave. HIV testing was offered to black participants in (only) the 2009 wave and 94% of the sample consented (see Additional file 1: Supplemental Digital Content 1 for details). We restricted the data to those reporting having ever had full penetrative sexual intercourse, as heterosexual transmission is the predominant mode of HIV transmission in the study setting. We also excluded individuals who self-reported being HIV positive at baseline (2005).

### Measures

Using data on HIV status from the HIV test conducted as part of the 2009 CAPS survey wave, we created a binary indicator for HIV-positive status (1 = yes) in 2009.

HIV-risk perceptions were assessed in the 2005 and 2009 waves using the question ‘Do you think you have no risk, a small risk, a moderate risk or a great risk of getting the AIDS virus?’ This survey instrument for risk perception has been used elsewhere [16, 22, 23]. Respondents had the option of reporting an HIV-positive status. “Don’t know” was included as a potential response. In our analysis of the accuracy of HIV risk perception, we use the 5-category (don’t know, no, small, moderate, or great risk) variable. For analysis of the correlates of risk perceptions in 2009, we created a binary variable to separate individuals who responded that they had no or small risk from those who perceived themselves at moderate or great HIV risk. Women answering “don’t know” were excluded from this measure. We justified this choice, which has precedent in the literature [11], on two grounds. First, only 73 respondents (12%) answered “don’t know.” Second, a “don’t know” response could reflect failure to disclose an HIV-positive status to study fieldworkers because of social desirability bias.

In analysing the correlates of risk perceptions, we used data on three sets of covariates, which were chosen a priori as factors that could theoretically influence risk perceptions or have been shown to do so in the literature. These covariates are all drawn from the 2009 data, as this survey wave contained the richest measures of demographic, sexual behaviour, and socio-psychological variables.

The first set of covariates comprised socioeconomic and demographic factors. Specifically, we considered measures of age, years of schooling, marital status, employment, and monthly household income per capita.

The second focused on current and historical sexual behaviours. We created a measure of condom use that identified individuals who reported consistently using condoms (always or usually) with their most recent partner. As multiple sexual partnerships are often associated positively with perceived HIV infection risk [22, 24]**,** we included a marker of having had three or more lifetime partners. (We chose this cut-off given that participants who did not provide an overall estimate of their number of life-time partners often reported at least three most recent partners in the survey’s partner history table.) We created a measure of age-disparate partnerships as those in which the male partner was 5 or more years older, which is consistent with the UNAIDS definition [25] and the literature [26, 27]. We also included binary indicators of previous sexually transmitted disease (STD) history, and whether or not women perceived their most recent partner to have other partners (following a growing literature around the effects of partner concurrency on self-perceived HIV risk) [11, 22, 24]. An indicator of any alcohol consumption over the previous month was used, as alcohol consumption has been associated with risky sexual behaviours [28]. It is worth noting that examining sexual behaviours is also a way to assess the validity of our measure of risk perceptions [29]. For example, a lack of correlation could signal a poor positive predictive value of the survey question, in addition to individual risk perceptions being inaccurate.

The third set of covariates focused on socio-psychological aspects relating to HIV, including beliefs and experiences. We created an indicator of HIV knowledge, based on a summation of correct responses to four questions (see Additional file 1: Supplemental Digital Content 2). We also included a binary indicator of beliefs about the protective benefits for men of male circumcision, following recent findings that women who believed that male circumcision protects men from HIV infection also believed the protection extended to women, and accordingly downgraded their perceived HIV risk [30, 31]. Such beliefs may be relevant in the context of our study, as the vast majority of black African men in the study region are circumcised [32]. HIV-related symbolic stigma (negative moral judgements of people living with HIV) may influence perceived risk if a blaming or ‘othering’ response leads to a heightened sense of invulnerability to HIV [33–35]. We created a measure of symbolic stigma based on the number of the following questions for which a stigmatising response was given: ‘Do you think HIV/AIDS is a punishment for sleeping around?’; ‘Do you think that some people with HIV/AIDS want to infect other people with the virus?’; ‘Do you think many people who get HIV infected through sex have only themselves to blame?’ To capture the effects of social networks, which have been shown in other work to be highly predictive of risk perceptions [28], we included a binary measure of knowing someone living with HIV or believed to have died of AIDS. Finally, we included binary measures of prior HIV testing and knowledge of partners’ HIV status.

### Analysis

First, we assessed the accuracy of perceived HIV risk. To do so, we identified all black women who were surveyed both in 2005 – the first year the perceived HIV-risk measure was fielded – and in 2009, the year when HIV tests were conducted. We used *χ* ^2^ tests to examine the association between respondents’ perceived risk in 2005 of contracting HIV and HIV test results in 2009. We excluded participants who reported an HIV-positive status in 2005 (*n* = 10), as it is not clear how HIV-positive women aware of their serostatus would have responded to the perceived risk question. We also analysed the association between perceived risk in 2009 of getting HIV and actual HIV status in 2009, in order to assess whether risk perceptions improved over time. To do so, we used all observations from the 2009 survey wave with the requisite information on both variables.

Second, we examined transitions in HIV risk perception between 2005 and 2009. Specifically, we calculated the HIV prevalence for each of four groups: those reporting low risk perceptions in both 2005 and 2009; those reporting high risk perceptions in both waves; and both transitions between high and low perceived risk. This analysis helps assess how risk perceptions evolve with age and whether the individuals adopt information relevant to their HIV risks over time. If, for example, individuals were correctly updating perceived HIV risk between survey waves on the basis of actual risk, we would expect to find higher HIV prevalence for individuals moving from low to high risk over that period than for those continuing to perceive low risks. We examined potential bias from attrition in the analysis of risk perception accuracy and transitions by assessing whether the probability of follow-up in 2009 was associated with perceived HIV-risk in 2005.

Third, we examined the predictors of perceived HIV risk in the 2009 CAPS data. We estimated the relationship between perceived HIV risk and the aforementioned covariates using multivariable logistic regression models. As discussed above, the choice of covariates was driven by a theoretical approach. (As a point of comparison, we conducted bivariate regressions, though these were not used as the basis for model selection.) Having excluded participants who self-reported being HIV-positive (*n* = 24), we conducted this analysis for the full sample. Last, we repeated the regression model using data only among those who tested HIV-negative in 2009. This latter model was motivated by the fact that women not wanting to disclose their HIV status to a fieldworker may have reported an HIV-negative status and provided data on their perceived risk of getting HIV in the future. That is, the second model removes potential bias introduced in the first analysis as a result of including individuals who knew they were HIV-positive but did not disclose this in the CAPS survey.

To account for non-response and complex survey design, we used CAPS sample weights and adjusted standard errors for clustering at primary sampling unit. All analyses were conducted with Stata 14.0 (Stata Corp. College Station, TX, USA).

## Results

### Sample characteristics

Descriptive statistics are presented in Table 1. Column 1 presents baseline (2005) data for the sample used for our prospective analysis of accuracy of perceived risk (*N* = 530). The average age was 21.3 years. Twenty-one percent had completed grade 12 and 40% were still enrolled in education. Few in the sample were married (6%) or employed (24%). Reflective of the high HIV prevalence in the region, more than two thirds knew someone living with HIV or who had died of AIDS (69%). Column 2 presents the characteristics of the cross-sectional sample (*n* = 539) used in the analysis of factors associated with perceived HIV risk in 2009. The average age was 24.8 years. Forty-two percent had completed grade 12 and 8% were still enrolled in education. Sixty-nine percent reported ever testing for HIV and 66% knew persons living with HIV or who had died of AIDS.

**Table 1:** Characteristics of study samples

	1 Baseline (2005) data on prospective cohort	2 2009 cross-sectional sample
	*N* = 530	*N* = 539
Age		
Mean (95% CI)	21.3 (21.1–21.5)	24.8 (24.6–25.0)
Years of education		
Mean (95% CI)	10.3 (10.1–10.5)	10.7 (10.5–10.9)
Completed grade 12	29%	42%
Enrolled in education	40%	9%
Marital status		
Married	6%	15%
Employment status		
Employed	24%	42%
Know someone with HIV or died of AIDS	69%	66%
HIV Knowledge		
Knows about MTCT	na	64%
Knows that a healthy looking person can have HIV	na	68%
Heard the circumcision protects men	na	26%
Number of stigmatising responses		
One	na	25%
Two or three	na	14%
HIV testing history		
Yes	na	69%
Know partner’s HIV status		
Yes	na	47%

Table provides weighted means (95% CI) and proportions for key sample characteristics (for the proportions, we do not report the number of individuals in each category given the use of sample weights). Column (1) contains baseline data for the sample (*N* = 530) used to analyse the accuracy of risk perceptions. This sample consists of all black, female respondents who reported risk perceptions in 2005 and who provided samples for HIV testing in 2009. Individuals who self-reported that they were HIV positive were excluded from the analysis samples. Column (2) contains baseline data for the sample (*N* = 539) used to assess correlates of risk perception. This sample includes all black women who provided data on perceived risk and on all covariates assessed. “Na” = survey question not asked in that year

### Association between perceived HIV risk and HIV test results

Among the cohort who provided an answer to the perceived risk question in 2005 *and* completed the HIV test in 2009, HIV prevalence in 2009 was 30%. More than two-thirds (69%) had perceived themselves at no HIV risk (33%) or small risk (36%), 22% at moderate/great risk and 9% were uncertain.

**Fig. 1:**
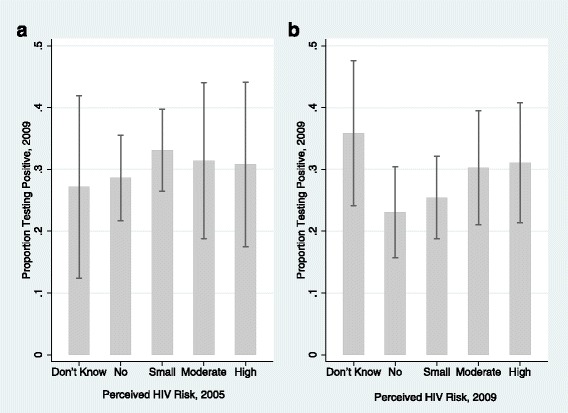
Association between perceived risk and HIV test results. Figures depict the proportions tested HIV positive in 2009 by self-perceived risk of getting HIV as reported in (**a**) 2005 (*n* = 530); and (**b**) 2009 (*n* = 649). Sample sizes for each figure reflect the largest number of individuals with available data

Figure 1a displays the proportion of individuals by perceived risk in 2005 who tested HIV positive in 2009. Women who had perceived themselves at no or small risk of getting HIV in 2005 were as likely to test positive for HIV in 2009 as individuals who had perceived themselves to be at moderate or great risk (31% vs 29%, *p* = 0.80), with the proportions ranging from 27 to 31%. Furthermore, the distribution of perceived risk responses in 2005 was extremely similar among individuals who tested HIV-negative compared to those who tested HIV-positive in 2009 (χ^2^ = 1.43; *p* = 0.85), which indicates that respondents were highly inaccurate at evaluating their own HIV risk.

Figure 1b displays results of the same analysis using the 2009 data. Among this sample (*n* = 649), the majority (55%) perceived themselves at no (24%) or small (31%) HIV risk, 33% at moderate/great risk and 12% were uncertain. Compared to the panel analysis (Fig. 1a), the proportion who tested HIV positive among those with a moderate/high perceived risk was very similar, while the HIV-positive percentage among women who perceived no/small HIV risk (24.4%) dropped. While this suggests that the accuracy of perceived risk improved somewhat over time, the difference in the proportion who were HIV positive between the no/small perceived risk group and the moderate/great perceived risk group remained small and statistically insignificant (24% vs 30%, *p* = 0.12). Furthermore the distribution of perceived risk responses in 2009 was not statistically different across HIV-positive and HIV-negative women (χ^2^ = 5.8; *p* = 0.24).

### Transitions in risk perceptions and HIV status

Table 2 presents HIV status in 2009 by changes in perceived risk between 2005 and 2009 among the panel sample (*n* = 446) of individuals with the perceived risk data in both years and who did not self-report being HIV positive in any survey. Approximately half (51%) reported a low perceived risk of contracting HIV in both 2005 and 2009. Almost a quarter (24%) of these women tested HIV-positive in 2009. Only 11% of the sample perceived themselves at high risk of contracting HIV in both 2005 and 2009. The difference in proportions of those who were HIV-positive among these women and those with low perceived risk in both years was small and not statistically significant (32% vs 24%, *p* = 0.31). Overall, the proportion testing HIV positive across all four transition categories was statistically similar (χ^2^ = 1.6; *p* = 0.68).

**Table 2:** Changes in perceived risk (2005–2009) and HIV status (2009)

	HIV- % (n)	HIV+ % (n)	Total % (n)
Perceived risk			
Low in 2005 and 2009	76 (172)	24 (57)	100 (229)
Low in 2005, high in 2009	71 (83)	29 (32)	100 (115)
High in 2005, low in 2009	74 (40)	26 (14)	100 (54)
High in 2005 and 2009	68 (32)	32 (16)	100 (48)
*χ* ^2^ (*p*-value)	1.60 (*p* = 0.68)		

Analysis denotes the proportion (n) testing HIV positive in 2009 (2 columns) by transitions in perceived risk status between 2005 and 2009 (4 rows)

Notably, we found no association between perceived risk in 2005 and attrition from CAPS between 2005 and 2009 (Additional file 1: Supplemental Digital Content 3), suggesting that differential attrition did not bias our findings.

### Predictors of perceived HIV risk

Table 3 presents the results from the multivariable logistic regression analysis examining correlates of self-perceived HIV risk reported in 2009 (bivariate estimates, which were, in general, substantively similar, are provided in Additional file 1: Supplemental Digital Content 4). In the full estimation sample (all available observations for African women - Column 1), three factors relating to sexual behaviour were substantively associated with perceived risk. Women who reported using condoms consistently with their most recent partner were less likely to perceive themselves at moderate/great risk (aOR: 0.57; 95% CI: 0.36, 0.89; *p* = 0.02). Women were more likely to perceive themselves at risk if they had had three or more lifetime partners (aOR: 2.37; 95% CI: 1.53, 3.73; *p* < 0.01) or their most recent partner was five or more years older, i.e. an age-disparate partnership (aOR: 1.73; 95% CI: 1.09, 2.76; *p* = 0.02). We found positive associations between perceived risk and both perceptions about partner concurrency and alcohol consumption, but these associations were small and not statistically significant. We found no association between risk perceptions and age. Several of the socio-psychological factors were associated with perceived risk. Women who had heard that male circumcision reduces a man’s HIV infection risk perceived themselves less likely to get HIV (aOR: 0.38; 95% CI: 0.22, 0.64; *p* < 0.01). A negative association was found between symbolic stigma and perceived risk: compared to women who did not report stigma on any item, those who reported stigma on one item had slightly lower odds of perceiving themselves at risk (aOR: 0.69; 95% CI: 0.37, 1.28; *p* = 0.24) and those that reported two or more stigma items had about half the odds of perceiving themselves at risk (aOR: 0.53; 95% CI: 0.26, 1.09; *p* = 0.09). Sensitivity analysis using a stigma score (a 0–12 scale based on summing 5-point Likert scale responses) confirmed a negative relationship (aOR: 0.91; 95% CI: 0.84, 0.99; *p* = 0.03).

**Table 3:** Correlates of moderate or greater self-perceived HIV risk

	(1) All respondents	(2) HIV-negative respondents
Demographics		
Age (years)	1.05	1.03
	(0.97, 1.14)	(0.94, 1.13)
Education (years completed)	0.96	0.95
	(0.82, 1.11)	(0.80, 1.12)
Married (ref: not married)	0.61	0.74
	(0.29, 1.29)	(0.30, 1.82)
Employed (ref: unemployed)	0.77	0.79
	(0.52, 1.15)	(0.48, 1.28)
Per capita monthly household income	1.00	1.00
	(1.00, 1.00)	(1.00, 1.00)
Sexual behaviour		
Condom used usually/always (ref: never/sometimes)	0.57**	0.65
	(0.36, 0.89)	(0.38, 1.11)
Lifetime partners ≥3 (ref: 1 or 2)	2.39***	2.68***
	(1.53, 3.73)	(1.54, 4.67)
Recent partner ≥5 years older (ref: <5 years older)	1.73**	1.32
	(1.09, 2.76)	(0.75, 2.35)
Had a sexually transmitted disease (ref: no)	0.89	1.00
	(0.52, 1.53)	(0.50, 2.02)
Recent partner perceived to have other partners (ref: no)	1.48	1.62
	(0.86, 2.53)	(0.88, 3.01)
Consumed alcohol in past 30 days (ref: no)	1.52	2.55**
	(0.84,2.78)	(1.21, 5.36)
Socio-psychological factors		
HIV knowledge score (0–4)	0.90	0.84
	(0.68, 1.19)	(0.60, 1.18)
Heard male circumcision reduces male HIV risk (ref: no)	0.38***	0.31***
	(0.22, 0.64)	(0.17, 0.59)
Stigmatising attitudes (ref: no stigma)		
Stigmatising response to 1 item	0.69	0.76
	(0.37, 1.28)	(0.37, 1.55)
Stigmatising response to 2/3 items	0.53*	0.47*
	(0.26, 1.09)	(0.20, 1.12)
Know someone living with HIV or who died of AIDS (ref: no)	2.27***	2.42***
	(1.36, 3.78)	(1.28, 4.60)
Ever tested for HIV (ref: no)	0.67*	0.23***
	(0.43, 1.07)	(0.13, 0.39)
Know partner’s HIV status (ref: no)	0.82	0.69
	(0.06, 11.10)	(0.39, 1.20)
Constant	2.27***	1.60
	(1.36, 3.78)	(0.07, 35.30)
Observations	539	374

Estimates of logistic regression models examining correlates of HIV risk perceptions. Dependent variable = 1 for moderate/high risk and zero for no or low risk. Data come from the 2009 wave of CAPS, and individuals answering “don’t know” to the HIV risk perceptions query are excluded (about 12% of respondents). Estimates reflect odds ratios, with 95% CI in parentheses. Sample weights, correcting for survey design and non-response, used in estimating all models. Column 1 is the model for all black female respondents. Column 2 focuses only on those testing negative for HIV in 2009

*** *p* < 0.01

** *p* < 0.05

* *p* < 0.1

Women who reported knowing someone living with HIV or who had died of AIDS were more likely to perceive themselves at risk (aOR: 2.27; 95% CI: 1.36, 3.78; *p* < 0.01). Women who reported knowing their partner’s HIV status were less likely to perceive themselves at moderate/great risk (aOR: 0.67; 95% CI: 0.43, 1.07; *p* = 0.09). Finally, previously having had a HIV test was associated with much lower odds of perceiving oneself to be at risk (aOR: 0.21; 95% CI: 0.13, 0.35; *p* < 0.01).

The analysis restricted to HIV-negative women (Table 3, column 2) found substantively similar associations between perceived HIV risk and having had three or more lifetime partners (aOR: 2.68; 95% CI: 1.54, 4.67; *p* < 0.01), hearing that male circumcision reduces male HIV risk (aOR:0.31; 95% CI: 0.17, 0.59; *p* < 0.01), reporting stigma on 2/3 items (aOR: 0.47, 95% CI: 0.20, 1.12; *p* = 0.09), knowing someone living with HIV or who had died of AIDS (aOR: 2.42; 95% CI: 1.28, 4.60; *p* < 0.01) and having had an HIV test (aOR: 0.23; 95% CI: 0.13, 0.39; *p* < 0.01).

## Discussion

In this study of young black African women in Cape Town, South Africa, we found no association between HIV risk perceptions and HIV test results four years later. Over 60% of respondents surveyed in 2005 and 2009 reported being at “no” or “small” risk, and half the sample perceived themselves to have a low HIV risk across both survey years. Approximately a quarter of the women perceiving themselves to have a low risk of HIV infection in each of these samples tested HIV positive. Strikingly, the proportion of women who reported a low perceived risk in 2005, but tested HIV positive four years later, was almost identical to the proportion who perceived a moderate or great risk of contracting HIV.

Consistent with prior work, perceived risks were associated with some – but not all – sexual behaviours [22]. Sexually transmitted disease history and perceived partner concurrency were only weakly correlated with risk perceptions. In addition, we found no association between age and risk perceptions, which was striking in this sample given the sharp increase in HIV risk between the ages of 20–30 among South African women [19]. In contrast, we found evidence that several psychosocial factors that are not directly linked to actual HIV risk influence women’s evaluation of their HIV risk, including prior (ostensibly negative) HIV test results, having heard that male circumcision is protective against HIV, and holding stigmatising beliefs. Collectively, the results suggest that the inaccuracy of HIV risk perceptions may arise from incomplete consideration of sexual risk factors and from the influence of a number of factors not directly related to sexual behaviour.

Our study has a number of limitations, many of which motivate future work. First, all of our data, except the HIV test result in 2009, were self-reported and may thus be affected by under-reporting or social desirability bias [36]. Related to this is the 12% of participants who answered “don’t know” when asked about risk perceptions in 2009, of whom over 30% tested positive for HIV. It is possible that these individuals knew about their HIV status at the time of the study interview and did not want to disclose their HIV status. It is also possible that HIV prevalence is in fact high among individuals uncertain about their risk of infection, which would add further evidence of the difficulty many individuals appear to have in assessing their HIV infection risk.

Second, the risk perception questions did not numerically specify what is meant by “small,” “moderate,” or “great” risk and therefore different individuals may have assigned different probabilities of future HIV infection to each of these categories. In addition, the question does not specify a time frame over which personal risk was to be evaluated. Consequently, it is possible that what appears to be inaccurate perception actually reflects poor discriminatory capacity in the survey instrument. While both the magnitude and direction of association between our sexual behaviour measures and risk perceptions supports the validity of our survey instrument [29], and other research using CAPS shows a concordance between changes in risk perceptions and sexual behaviours within the same individual [5], it remains possible that noise in the survey measure could bias estimates of its predictive power to the null [37].

Third, given high rates of incidence and prevalence in the age group we studied, it is possible that risk perceptions may more accurately predict *the timing* of disease acquisition, even if they do not predict HIV status at a specific time point four years later. Unfortunately, we do not have information on HIV test results prior to the 2009 study wave and therefore cannot completely rule out this possibility. However, we do have risk perception measures at multiple time points. The fact that these measures show no relationship with HIV test results both one and four years out – along with the fact that transitions across risk perception categories were uncorrelated with HIV risk – provides further evidence of their inaccuracy.

Fourth, while our analysis was intended to be descriptive, it deserves mention that our findings cannot be construed as causal relationships, given the possibility of a number of unmeasured confounders. Fifth, our results are based on a sample from a single metro area, drawn from the end of the prior decade. As such, the applicability of our results to other contexts is not obvious. That said, our findings do apply to a very high-risk population in a country with one of the largest HIV/AIDS burdens in the world, thus giving them intrinsic value. Moreover, recent work from South Africa suggests a similar fraction of individuals self-reporting no or low risk of contracting HIV [22] – suggesting that our findings may have contemporary relevance. Regardless, few (if any) datasets from the study region contain as rich information on risk perceptions, sexual behaviour, and psychosocial measures as does CAPS.

Our results have several important implications. From a programmatic perspective, successful implementation of programs premised on the UNAIDS 90–90-90 target may depend greatly on the accuracy of risk perceptions. This supposition is supported by a growing literature linking risk perceptions to risky behaviours and likelihood of HIV testing. Thus, our findings of a mismatch between HIV risk perceptions and actual risk, particularly in a population where actual risk is generally underestimated, point to the need for interventions to improve the accuracy of risk perceptions. The importance of such interventions is highlighted by recent evidence from South Africa demonstrating continued shortfalls in HIV testing rates, despite aggressive implementation of a nationwide testing campaign [38].

Our analysis of the determinants of risk perception offers insights into how risk perceptions may be modified. In terms of magnitude, the factor most strongly correlated with risk perceptions in our data was prior HIV testing, which reduced the odds of reporting moderate or great risk five-fold. Given the young age of our sample and given that we excluded women who self-reported having HIV, it is likely that the majority of women previously tested had received a negative test result. Accordingly, our finding suggests that learning that one does not have HIV may significantly impact perceptions of future risk. This result calls for explicit counselling around the meaning of a negative HIV test and the sources of potential on-going HIV-infection risk. Specifically, a component of counselling should be aimed at countering the tendency for participants to downgrade their perceived risk after a negative result. Our findings also point to the need for public education about the fact that male circumcision affords no direct protection to women [30, 31]. Messages aiming to educate women that having a circumcised partner does not reduce personal HIV-infection risk would be important both in populations where male circumcision is typically conducted as part of traditional rituals, as is the case in our study, and populations who use voluntary medical male circumcision services. More generally, these findings underline the need for the provision of more granular, age and sexual-behaviour specific information on HIV risk, which in other populations has been shown to reduce risky sexual behaviours [39].

The link between stigmatising attitudes towards people living with HIV and lower HIV risk perceptions is troubling as stigma remains a common phenomenon in sub-Saharan Africa [40, 41]. Our finding is consistent with previous research [42], and supports the hypothesis that individuals who are prone to HIV-related symbolic stigma (negative moral judgements of people living with HIV) may perceive themselves as different from those with HIV and consequently perceive themselves as being less vulnerable to infection [34]. This result adds to the large body of evidence on the damaging effects of stigmatising attitudes on other health related perceptions and behaviours and highlights the importance of continued efforts to reduce stigma. Interventions to improve risk perceptions should, additionally, be cognizant that some individuals will hold stigmatising attitudes and perceive HIV risk to be outside their domain, and will therefore need to be designed specifically to counter this perception.

While it is clearly important to align perceived HIV-infection risk with actual HIV-infection risk, the above discussion suggests that interventions to do so may require a good deal of time to change relevant social attitudes and norms. Our results suggest that, in the short run, policymakers may also need to deploy interventions that do not rely on accurate risk perceptions. Such interventions include ‘opt-out’ programs, which may help to divorce risk perceptions from program take-up.

## Conclusions

Our results indicate that HIV risk perceptions among black South African women are highly inaccurate, likely due to the influence of factors not directly related to sexual behaviour. Findings imply that the uptake of existing HIV services may be suboptimal if they rely on accurate evaluations of perceived risk, and point to the need for interventions that modify perceived HIV-risk.

## Additional file

Additional file 1: Supplemental Digital Content 1; Supplemental Digital Content 2; Supplemental Digital Content 3 and Supplemental Digital Content 4. (DOCX 42 kb)
