# Supplementary material for: Accuracy and determinants of perceived HIV risk among young women in South Africa
Source: BMC Public Health. 2017 Jul 21;18:42. doi: 10.1186/s12889-017-4593-0 (PMC5520344; doi:10.1186/s12889-017-4593-0)
Supplement: Additional file 1: — Supplemental Digital Content 1; Supplemental Digital Content 2; Supplemental Digital Content 3 and Supplemental Digital Content 4. (DOCX 42 kb) [file 12889_2017_4593_MOESM1_ESM.docx]

**Supplemental Digital Content**

Accuracy and determinants of perceived HIV risk among young women in South Africa

**Supplemental digital content 1: CAPS Wave 5 HIV testing protocol**

On completion of the questionnaire the African respondents were asked to participate in the HIV testing component of the study by giving a DBS (preferably) or saliva (if hesitant about a finger prick) specimen. Respondents received a schedule of dates and locations for the Tutu Tester (a general-health mobile clinic) where they could get their HIV test result – results were identified by a barcode and emailed from the testing laboratory. Global Clinical & Viral Laboratory (accredited by the South African National Accreditation Services) managed the specimens, tested for HIV and compiled the HIV-status database. Specimens collected by the fieldworkers were returned to the fieldwork manager, logged onto a laboratory tracking sheet, and couriered to the laboratory within seven days. HIV tests were done using HIV antibody enzyme-linked immunosorbent assays (ELISAs). The testing strategy displayed in Figure 1 was used:

- A1. Screening assay: Vironostika Uniform 11 Plus O ( HIV-1p24; HIV-1gp160;HIV-1ANT70; HIV-2 env peptide amino acids 592-603), Biomeriux, Boxtel
- A2. Second tests: SD Bioline (HIV-1 gp41 including Subtype O, p24, HIV-2 gp36) 3^rd^ generation Standard Diagnostics Onc, Korea
- A3. Third tests: Western Blot (HIV1/2 Biorad Western Blot)

Figure - CAPS Wave 5 HIV-testing algorithm


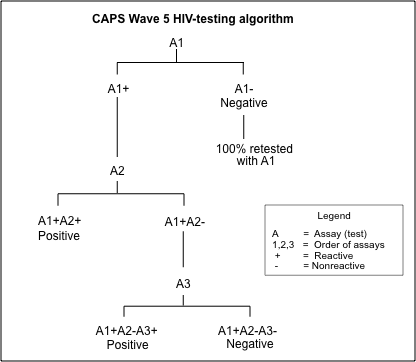


More specifically, the standard algorithm used two ELISAs based on different antigens. The first (A1) screened samples as either positive or negative. The negative specimens were pooled in groups of four and retested for HIV using A1. If negative, all four specimens were considered HIV negative. If the test on the pooled specimens was positive, each individual specimen was retested to determine which one was indeterminate. In practice this meant that all negative specimens were retested. All positive samples were retested using the second ELIZA (A2). Discordant samples that were positive in the first test and negative in the second were classified as ‘indeterminate’. These ‘indeterminates’ were subjected to a western blot confirmatory test (A3), the result of which was considered final for the indeterminate samples.

**Supplemental Digital Content 2 -** Relevant survey questions from the Cape Area Panel Study

Perceived risk

- Do you think you have no risk, a small risk, a moderate risk or a great risk of getting the AIDS virus?

STD History

- Have you ever had a sexually transmitted disease (STD) that is not HIV?

Partner concurrency

- As far as you know, did [**partner**] have any other sexual partners during the time that you and he/she were having a sexual relationship?

Alcohol consumption

- Over the past month, have you consumed any alcohol?

HIV knowledge

- Do you think you can get HIV/AIDS by eating food prepared by someone with HIV/AIDS?
- Do you think you can get HIV/AIDS by being coughed or sneezed on by someone who has HIV/AIDS?
- Can HIV/AIDS be transmitted from a mother to her child?
- Is it possible for a healthy-looking person to have HIV?

Male circumcision and HIV risk

- Have you ever heard that removing a man’s foreskin reduces the risk of him getting HIV?

HIV-related stigmatising attitudes

- Do you think HIV/AIDS is a punishment for sleeping around?
- Do you think that many people who get HIV infected through sex have only themselves to blame?
- Do you think that some people with HIV/AIDS want to infect other people with the virus?

**Supplemental Digital Content 3 –** Multivariable logistic regression of the determinants of survey attrition between wave 3 (2005) and wave 5 (2009)

| Dependent variable: |  |
| --- | --- |
| 0=interviewed in 2005 & 2009  1=not re-interviewed in 2009 | All respondents |
|  | aOR (95%CI) |
| Perceived HIV risk (0=no/small risk) | 1.08 |
|  | (0.66, 1.75) |
| Age | 1.01 |
|  | (0.90, 1.12) |
| Education (years completed) | 1.11 |
|  | (0.97, 1.27) |
| Married | 2.08* |
|  | (0.90, 4.79) |
| Employed | 1.27 |
|  | (0.77, 2.09) |
| Condom used at last sex | 1.28 |
|  | (0.79, 2.08) |
| Lifetime partners ≥ 3 | 1.17 |
|  | (0.74, 1.87) |
| Ever pregnant | 0.95 |
|  | (0.59, 1.54) |
| Recent partner perceived to have other partners | 1.12 |
|  | (0.72, 1.72) |
| Consumed alcohol in past 30 days | 1.22 |
|  | (0.68, 2.22) |
| Know someone with HIV | 1.19 |
|  | (0.73, 1.96) |
| Know someone who died of HIV | 0.71 |
|  | (0.43, 1.18) |
| Observations | 539 |

Notes: Estimates reflect odds ratios (95% CI). See main text for variable definitions. *** p<0.01, ** p<0.05, * p<0.1.

**Supplemental Digital Content 4 –** Bivariate analyses of factors associated with self-perceived HIV risk in 2009 among 20-30 year-old black African women..

|  | All respondents (N=539) |
| --- | --- |
|  | OR (95%CI) |
| **Demographics** |  |
| Age (years) | 1.03 |
|  | (0.96 - 1.10) |
| Education (years completed) | 0.92 |
|  | (0.80 - 1.05) |
| Married (ref: not married) | 0.49** |
|  | (0.27 - 0.89) |
| Employed (ref: unemployed) | 0.79 |
|  | (0.56 - 1.14) |
| Per capita monthly household income | 1.00** |
|  | (1.00 - 1.00) |
| **Sexual behaviour** |  |
| Condom used usually/always (ref: never/sometimes) | 1.00 |
|  | (0.66 - 1.52) |
| Lifetime partners ≥ 3 (ref: 1 or 2) | 2.42*** |
|  | (1.67 - 3.51) |
| Recent partner ≥ 5 years older (ref: <5 years older) | 1.35 |
|  | (0.92 - 1.99) |
| Had a sexually transmitted disease (ref: no) | 0.91 |
|  | (0.58, 1.43) |
| Recent partner perceived to have other partners (ref: no) | 1.61* |
|  | (0.98, 2.65) |
| Consumed alcohol in past 30 days (ref: no) | 1.55* |
|  | (0.93, 2.56) |
| **Socio-psychological factors** |  |
| HIV knowledge score (0-4) | 0.81* |
|  | (0.63, 1.04) |
| Heard male circumcision reduces male HIV risk (ref: no) | 0.73 |
|  | (0.45, 1.17) |
| Stigmatising attitudes (ref: no stigma) |  |
| Stigmatising response to 1 item | 0.63* |
|  | (0.39, 1.02) |
| Stigmatising response to 2/3 items | 0.43** |
|  | (0.23, 0.82) |
| Know someone living with HIV or who died of AIDS | 2.06*** |
|  | (1.34, 3.18) |
| Ever tested for HIV (ref: no) | 0.25*** |
|  | (0.17, 0.38) |
| Know partner’s HIV status (ref: no) | 0.59*** |
|  | (0.39, 0.87) |

**Notes**: Each cell represents results from a separate logistic regression. Estimates reflect (unadjusted) odds ratios with 95% CI in parentheses. Sample weights, correcting for survey design and non-response, used in all models. Estimation sample is the same as that used in Table 3 of the main text.
